# A Multisensory fMRI Investigation of Nociceptive-Preferential Cortical Regions and Responses

**DOI:** 10.3389/fnins.2021.635733

**Published:** 2021-04-14

**Authors:** Xiaoxia Zhang, Linling Li, Gan Huang, Li Zhang, Zhen Liang, Li Shi, Zhiguo Zhang

**Affiliations:** ^1^Health Science Center, School of Biomedical Engineering, Shenzhen University, Shenzhen, China; ^2^Guangdong Provincial Key Laboratory of Biomedical Measurements and Ultrasound Imaging, Shenzhen, China; ^3^Marshall Laboratory of Biomedical Engineering, Shenzhen University, Shenzhen, China; ^4^Peng Cheng Laboratory, Shenzhen, China

**Keywords:** nociception, pain, fMRI, multisensory, sensory perception

## Abstract

The existence of nociceptive-specific brain regions has been a controversial issue for decades. Multisensory fMRI studies, which examine fMRI activities in response to various types of sensory stimulation, could help identify nociceptive-specific brain regions, but previous studies are limited by sample size and they did not differentiate nociceptive-specific regions and nociceptive-preferential regions, which have significantly larger responses to nociceptive input. In this study, we conducted a multisensory fMRI experiment on 80 healthy participants, with the aim to determine whether there are certain brain regions that specifically or preferentially respond to nociceptive stimulation. By comparing the evoked fMRI responses across four sensory modalities, we found a series of brain regions specifically or preferentially involved in nociceptive sensory input. Particularly, we found different parts of some cortical regions, such as insula and cingulate gyrus, play different functional roles in the processing of nociceptive stimulation. Hence, this multisensory study improves our understanding of the functional integrations and segregations of the nociceptive-related regions.

## Introduction

Pain is an unpleasant sensory and emotional experience associated with actual or potential tissue damage or described in terms of such damage (IASP, International Association for the Study of Pain) (Loeser and Treede, 2008). By using neuroimaging methods, such as functional magnetic resonance imaging (fMRI), electroencephalography (EEG), and positron emission tomography (PET), we can detect brain activities and cortical regions that are induced by pain perception (Tölle et al., 1999; Iannetti et al., 2005; Moisset and Bouhassira, 2007; Wager et al., 2013; Jackson et al., 2020). More specifically, it has been well-documented that nociceptive pain (Mischkowski et al., 2018), which is caused by noxious stimuli in intact tissue, can trigger brain activities in a network of cortical regions, including the thalamus, insula, the primary and secondary somatosensory cortices, and the cingulate cortex, which are often called “pain matrix” (Ingvar, 1999; Peyron et al., 2000, 2002; Apkarian et al., 2005).

However, whether the pain matrix is specific to pain (i.e., activated by pain stimulation only) has long been a controversial issue. Some pain researchers believe that at least part of the pain matrix is specific to nociceptive pain (Stern et al., 2006; Tracey and Mantyh, 2007; Boly et al., 2008; Borsook et al., 2010), but more evidence have shown that almost all cortical regions in the pain matrix can be activated by other sensory modalities (Downar et al., 2000; Macaluso and Driver, 2005; Lui et al., 2008; Mouraux and Iannetti, 2009; Mouraux et al., 2011). Some multisensory studies, which used a random sequence of different types of sensory stimulation to examine activated cortical regions, found that non-nociceptive somatosensory, auditory, and visual stimulation can activate cortical regions largely similar to those regions activated nociceptive somatosensory stimulation (Downar et al., 2000; Mouraux and Iannetti, 2009; Mouraux et al., 2011). Based on this phenomenon, many researchers put forward another possible point of view: the so-called “pain matrix” may be related to the significant input of sensory stimulation and the ability to respond to it, regardless of the type of sensory stimulation input (Iannetti and Mouraux, 2010; Legrain et al., 2011; Mouraux et al., 2011; Iannetti et al., 2013). In another word, the pain matrix is not specific to pain.

However, these multisensory studies were based on a relatively small sample size (the number of participants was smaller than 20), and the statistical power of the results was weak. Another important limitation of existing multisensory studies concerning the nociceptive pain is that, they were focused on the identification of nociceptive-specific regions, but overlooked the possibility that some cortical regions may be preferential to nociceptive pain, even they are not specific to nociceptive pain. Nociceptive-preferential regions are cortical regions that can be evoked by stimulation of various types of sensory modalities but are more preferentially activated by nociceptive stimulation. In another word, nociceptive-preferential regions may have significantly greater response to nociceptive pain rather than to other sensory modalities. However, the existence of nociceptive-preferential regions has been seldom investigated by a multisensory study. It is important to identify and differentiate nociceptive-specific and nociceptive-preferential cortical regions to improve our understanding of the functional integrations and segregations of these regions.

In this study, we hypothesized that at least some cortical regions are preferentially involved in the processing of nociceptive input and validated our hypothesis based on fMRI data recorded from a relatively large number of participants (80 healthy participants) in a multisensory experiment. We applied a random sequence of stimuli with four different sensory modalities, including nociceptive somatosensory, non-nociceptive somatosensory, auditory, and visual, on these participants, and recorded the fMRI responses. We used the conjunction analysis on the evoked fMRI activities to identify cortical regions that are specifically activated by one type of sensory modality (i.e., modality-specific brain regions). We further compared evoked fMRI magnitudes among four sensory modalities to identify cortical regions that are preferentially activated by one specific sensory modality (i.e., modality-preferential brain regions). These identified nociceptive-specific and -preferential regions were further compared to check their overlap and difference.

## Materials and Methods

### Participants

Eighty healthy right-handed participants aged 21.0 ± 3.1 years, with 36 men and 44 women, were recruited. All participants were healthy and had no neurological or cardiovascular diseases or neurological disorders or acute/chronic pain. The study was approved by the local Ethics Committee and all participants gave written informed consent.

### Experiment

Each participant received four types of sensory modality: nociceptive somatosensory, non-nociceptive somatosensory, auditory, and visual, presented in a random order with similar attentional level. Nociceptive somatosensory stimuli were infrared neodymium yttrium aluminum perovskite (Nd: YAP) laser pulses delivered to the left-hand dorsum. The laser pulse duration was approximately 4 ms, and the spot size was 7 mm in diameter. Non-nociceptive somatosensory stimuli were bipolar square wave electrical pulses stimulation (100 Hz frequency, 1,000 μs pulse width, single pulse, forward current, and 1 ms duration). The stimuli were delivered through a pair of skin electrodes (1 cm inter-electrode distance) placed at the inside of the left arm, over the median nerve. Auditory stimuli adopted binaural monophonic stimulation (800 Hz auditory tones lasting 50 ms, 5 ms rise and fall times) and delivered through pneumatic earphones. Visual stimuli consisted of a bright white disk displayed on the projection screen for 50 ms. For each sensory modality, two stimulus intensity levels were used: 3 and 3.5 J for nociceptive somatosensory, 2 and 4 mA for non-nociceptive somatosensory, 76 dB SPL and 88 dB SPL for auditory stimuli, two different grayscale images for visual stimuli.

The experiment consisted of two runs. Each run consisted of 4 types of modality stimuli, and each was delivered 20 times in a pseudo-random order, 10 times for each intensity level. A white fixation cross was presented for 600 s at the center of the black screen, and the stimulus was delivered in the meantime. Ten seconds after one stimulus, the participant was required to rate the intensity of the stimulus received using a visual analog scale (VAS) ranging between 0 (not perceived) and 10 (maximum intensity) within 5 s. The interval between each stimulus is 22–23 s, and each run lasted for about 15 min.

fMRI data were acquired using a standard gradient echo-planar imaging sequence by a GE 3.0T MRI scanner with the following parameters: 43 axial slices, thickness/gap = 3/0 mm, time of echo (TE) = 30 ms, time of repetition (TR) = 2,000 ms, acquisition matrix = 64 × 64, field of view (FOV) = 192 × 192 mm^2^, flip angle (FA) = 90°, acquisition time = 30:12 min. A total of two sessions with 454 functional volumes were collected in the multisensory stimulation task. At the end of the task experiment, a high resolution T1-weighted structural image was collected for spatial registration with the following parameters: 176 sagittal slices, *TE* = 2.992 ms, *TR* = 6.896 ms, inversion time *T1* = 450 ms, 1 mm slice thickness with no gap, 1 × 1 mm in-plane resolution, acquisition matrix = 256 × 256, acquisition time = 4:36 min.

### fMRI Data Preprocessing

fMRI data were preprocessed using Statistical Parametric Mapping (SPM12)^1^. The first three volumes were removed for signal equilibration, and 451 volumes were remained. Functional images were slice-time corrected, motion-corrected (realigned), coregistered to the structural images, spatially normalized to the Montreal Neurological Institute (MNI) template, resampled to 3 × 3 × 3 mm voxel size, spatial smoothed using a full-width at half-maximum (FWHM) Gaussian kernel of 8 mm, and then high-pass temporally filtered (1/128 Hz cutoff).

### General Linear Model

The task activated fMRI patterns were analyzed using the General Linear Model (GLM) in SPM. GLM was performed in two levels. The first-level analysis was performed- for each participant with regressors modeling the occurrence of each condition (nociceptive somatosensory, non-nociceptive somatosensory, auditory, visual, and the rating period) and six additional regressors of head motion parameters. Four contrast analyses corresponding to the four stimulus types were performed to assess the BOLD responses of each participant and to produce a statistical parametric map for each participant. The first-level statistical parametric maps of all participants were then entered into a second-level one-sample *t*-test analysis for each stimulation. The second-level statistical results were further performed by the Benjamini-Hochberg procedure to decrease the False Discovery Rate (FDR) and the corrected significance level was set to 0.0001. We also used other significance thresholds to examine the robustness of the results (for example, results obtained with *P* < 0.05 with FDR correction can be found in Supplementary Figure 1).

### Identification of Modality-Specific Regions (Conjunction Analysis)

To identify brain regions specific to each type of stimulation (i.e., activities only elicited by one sensory modality, but not by other three), we performed a conjunction analysis based on the Minimum Statistic compared to the Conjunction Null (MS/CN) method (Nichols et al., 2005). We calculated the intersection of four modalities’ statistical maps, which were obtained by GLM to identify multimodal regions, i.e., those brain regions co-activated by all four modalities. And the brain regions specific to one specific sensory modality can be identified as those regions that were activated by only this sensory modality but not by others. All the identified voxels were defined using the automatic anatomical labeling (AAL) atlas (Collins et al., 1998), and the threshold of cluster size was set to 20 (i.e., only a cluster with more than 20 voxels was retained).

### Identification of Modality-Preferential Regions (PSC Analysis)

Further, in order to identify modality-preferential regions (i.e., those regions are preferentially activated by one specific sensory modality), we extracted percentage signal change (PSC) at each voxel (Pernet, 2014). PSC features were used here because PSC can more accurately fit the fMRI data and compensate temporal shift than GLM-based coefficients (Calhoun et al., 2004). PSC was calculated as the ratio between the magnitude of the BOLD response accounting for the temporal shift and the mean of the adjusted time series. Before extracting PSC features, preprocessed fMRI data (see section “fMRI Data Preprocessing”) were further processed with the following extra steps: (1) linearly detrending the time-series data to eliminate signal drift caused by factors such as machine heat and pulse, (2) regressing out six motion parameters and the rating regressor modeling the occurrence of the rating period.

Specifically, for each sensory modality (i.e., nociceptive somatosensory, non-nociceptive somatosensory, auditory, and visual), we extracted time-series data according to the stimulus onset for each trial at each voxel, and then we calculated the PSC for each stimulus, as described previously. We then averaged the PSC cross trials for each sensory modality. Then, we obtained the average PSC values of each voxel for each sensory modality and each subject.

To identify voxels that are preferentially activated by one certain sensory modality, we compared extracted PSC features among sensory modalities using a voxel-wise one-way repeated measures analysis of variance (ANOVA), followed by *post-hoc* Tukey-Kramer comparisons. To address the multiple comparisons problem, we computed the Benjamini-Hochberg correction of the FDR and set the corrected significance level to 0.0001. To examine the robustness of the results, brain regions identified using *P* < 0.05 with FDR correction are shown in Supplementary Figure 2. For each voxel, if its average PSC of one sensory modality was significantly larger than the average PSCs of other three sensory modalities, then this voxel was considered as “preferentially” activated by this sensory modality. Above analysis was performed for each sensory modality and we finally had four modality-preferential maps for four types of sensory stimulation. All the identified voxels were defined using the AAL atlas (Iannetti et al., 2013), and the threshold of cluster size was set to 20.

Further, we examined the across-trial correlation between subjective rating and PSCs in the modality-preferential regions for each sensory modality. For each subject, the PSCs of each trial were averaged across all voxels within the modality-preferential regions. We then calculated Pearson’s correlations between PSCs and VAS ratings across trials for each subject. The obtained correlation coefficients for each subject were then normalized by Fisher’s z-transformation, and one-sample *t*-test was performed to determine whether there was a significant cross-subject correlation between the VAS rating and PSCs for each sensory modality.

## Results

### Behavioral Results

**FIGURE 1:**
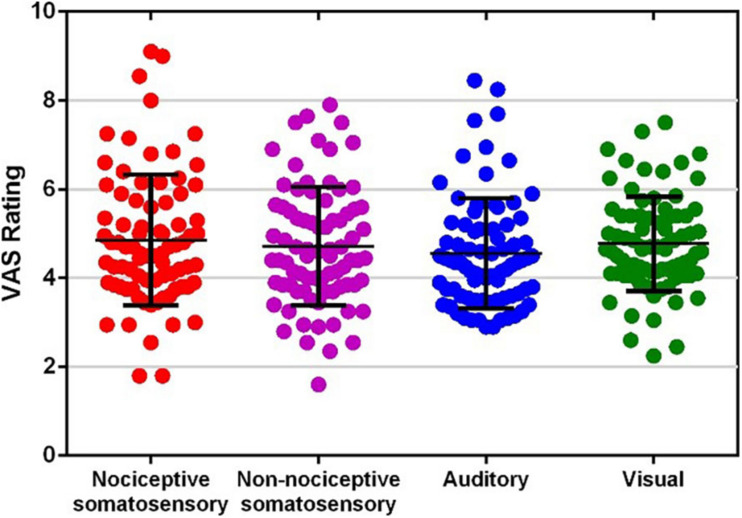
Visual analog scale (VAS) ratings of each type of sensory modality. Each dot represents the average rating of one participant to one type of sensory modality.

Figure 1 shows the VAS ratings of each type of sensory modality. The average VAS rating for each sensory modality was (mean ± SD): nociceptive somatosensory: 4.858 ± 1.472; non-nociceptive somatosensory, 4.715 ± 1.335; auditory: 4.550 ± 1.241; visual: 4.771 ± 1.061. There was no significant difference among the VAS ratings of four sensory modalities (F (2.655, 209.752) = 1.936, *P* = 0.132, one-way repeated measures ANOVA).

### General Linear Model Analysis

**FIGURE 2:**
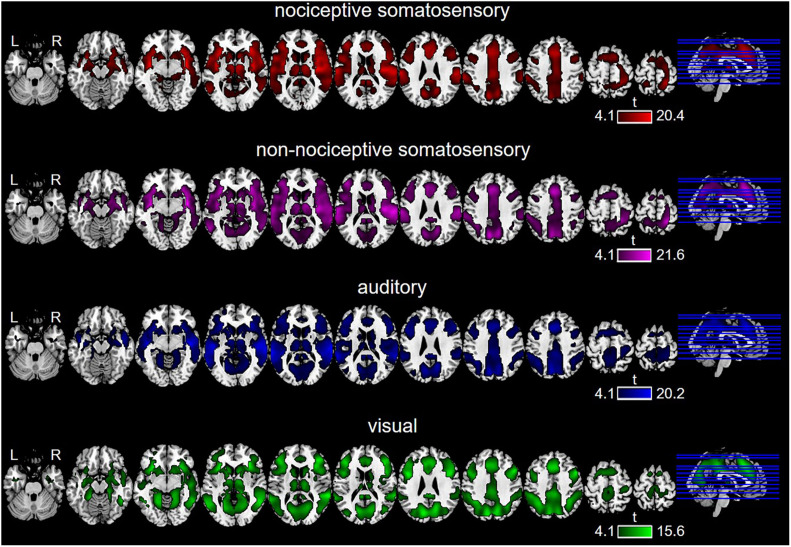
Brain regions activated by each type of sensory stimulation, as revealed by General Linear Model (GLM) (*P* < 0.0001, FDR-corrected).

The GLM-based activated regions are shown in Figure 2. Nociceptive somatosensory, non-nociceptive somatosensory, auditory, and visual stimuli elicited widely distributed fMRI brain activities throughout the brain regions. Besides, the considerable overlaps among the brain regions activated by different sensory modalities were noticeable.

**FIGURE 3:**
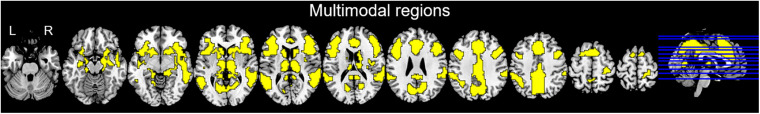
Binary multimodal regions defined by conjunction analysis.

Multimodal regions (i.e., those brain regions co-activated by nociceptive somatosensory, non-nociceptive somatosensory, auditory, and visual stimuli) on wide-field included the prefrontal cortex, sub-lobar (mainly including the thalamus, caudate, and putamen), temporal lobe (mainly including superior temporal gyrus, middle temporal gyrus), insula, cingulate gyrus, precuneus, inferior parietal lobule, precentral gyrus, and cuneus (Figure 3).

### Modality-Specific Regions Identified by Conjunction Analysis

**FIGURE 4:**
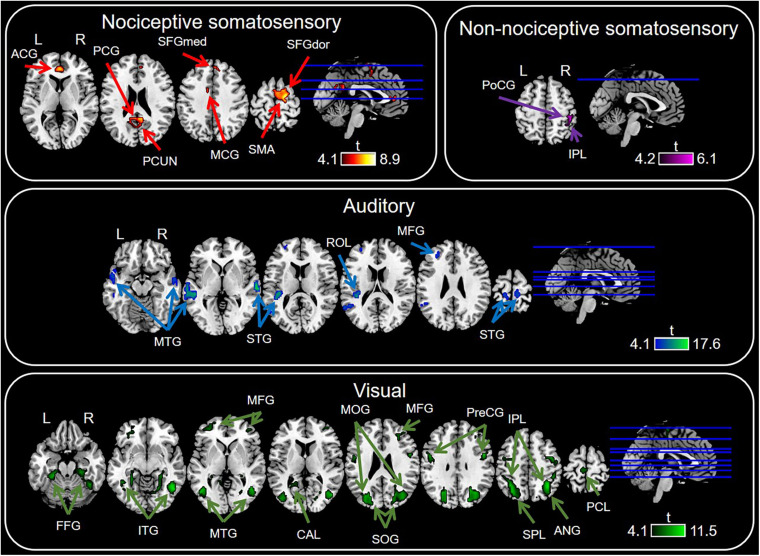
Sensory modality-specific regions defined by conjunction analysis of General Linear Model (GLM) maps. Significant results were identified at *P* < 0.0001 with FDR correction. ACG, anterior cingulate gyrus; ANG, angular gyrus; CAL, calcarine sulcus; MCG, middle cingulate gyrus; FFG, fusiform gyrus; IPL, inferior parietal lobule; ITG, inferior temporal gyrus; MFG, middle frontal gyrus; MOG, middle occipital gyrus; MTG, middle temporal gyrus; PCG, posterior cingulate gyrus; PCL, paracentral lobule; PCUN, precuneus; PoCG, postcentral gyrus; ROL, rolandic operculum; PreCG, precentral gyrus; SFGdor, dorsolateral part of superior frontal gyrus; SFGmed, medial part of superior frontal gyrus; SMA, supramarginal gyrus; SOG, superior occipital gyrus; SPL, superior parietal lobule; STG, superior temporal gyrus.

Conjunction analysis in specific sensory modality showed that there were some brain regions specifically elicited by nociceptive somatosensory, non-nociceptive somatosensory, auditory, or visual stimuli at *P* < 0.0001 with FDR correction (Figure 4 and Table 1), while *P* < 0.05 for the specific brain regions see Supplementary Figure 1. Nociceptive somatosensory-specific regions were mainly in the ipsilateral and contralateral cingulate gyrus, the ipsilateral and contralateral precuneus, and the contralateral frontal lobe (including the supplementary motor area, dorsolateral and medial part of superior frontal gyrus). Brain responses activated by stimulation of non-nociceptive somatosensory were mainly in the contralateral postcentral and inferior parietal lobule. Auditory-specific regions were mainly in the temporal gyrus, and there was also a small number of activations in the frontal gyrus, paracentral lobule, rolandic operculum, postcentral gyrus and supramarginal gyrus. Visual-specific regions were mainly in the occipital lobe, extending into the temporal and parietal lobe, and also in the precentral and postcentral gyrus.

**TABLE 1:** List of Modality-specific brain regions as revealed by General Linear Model (GLM).

Effect	Left hemisphere				Right hemisphere			
		
Region	x	y	z	No. of voxels	x	y	z	No. of voxels
**Nociceptive-somatosensory**								
SFGdor, dorsolateral part of superior frontal gyrus	−	−	−	−	21	−12	63	55
SMA, supplementary motor area	0	3	57	16	15	−9	66	125
SFGmed, medial part of superior frontal gyrus	−	−	−	−	6	45	33	25
ACG, anterior cingulate gyrus	−	−	−	−	9	45	30	10
ACG, anterior cingulate gyrus	0	42	6	18	3	42	6	6
MCG, middle cingulate gyrus	−6	3	42	6	−	−	−	−
PCG, posterior cingulate gyrus	−3	−48	21	23	−	−	−	−
PCUN, precuneus	−3	−63	27	22	9	−48	21	41
**Non-nociceptive-somatosensory**								
PoCG, postcentral gyrus	−	−	−	−	42	−30	57	20
IPL, inferior parietal lobule	−	−	−	−	45	−36	57	11
**Auditory**								
STG, superior temporal gyrus	−45	−33	6	56	63	−15	3	32
MTG, middle temporal gyrus	−51	−33	6	164	−	−	−	−
MTG, middle temporal gyrus	−60	−54	18	22	66	−24	−3	35
SFGdor, dorsolateral part of superior frontal gyrus	−21	54	6	9	−	−	−	−
MFG, middle frontal gyrus	−30	42	24	29	−	−	−	−
PCL, paracentral lobule	−175	−33	69	14	9	−39	69	31
ROL, rolandic operculum	−36	−36	18	21	−	−	−	−
PoCG, postcentral gyrus	−18	−33	63	9	−	−	−	−
SMG, supramarginal gyrus	−54	−48	30	14	−	−	−	−
TPOsup, superior temporal pole	−48	6	−18	12	−	−	−	−
**Visual**								
CAL, calcarine sulcus	−24	−54	9	21	18	−78	18	14
LING, lingual gyrus	−	−	−	−	21	−57	−9	24
SOG, superior occipital gyrus	−24	−66	36	76	31	−63	39	114
MOG, middle occipital gyrus	−24	−63	39	184	33	−63	36	152
FFG, fusiform gyrus	−21	−39	−15	60	21	−33	−15	83
SPL, superior parietal lobule	−27	−60	45	111	33	−60	51	12
IPL, inferior parietal lobule	−27	−54	42	77	33	−51	45	63
SMG, supramarginal gyrus	−	−	−	−	45	-36	45	7
ANG, angular gyrus	−	−	−	−	36	−54	48	63
PCUN, precuneus	−15	−45	3	8	24	−60	27	14
MTG, middle temporal gyrus	−45	−57	−3	22	45	−57	3	87
ITG, inferior temporal gyrus	−45	−54	−6	7	48	−48	−15	95
ORBsup, orbital part of superior frontal gyrus	−24	60	0	12	−	−	−	−
MFG, middle frontal gyrus	−	−	−	−	30	33	24	23
MFG, middle frontal gyrus	−36	51	6	22	42	48	3	18
ORBmid, orbital part of middle frontal gyrus	−27	42	−9	15	−	−	−	−
ORBinf, orbital part of inferior frontal gyrus	−36	42	−3	10	39	45	0	6
PCL, paracentral lobule	−3	−21	66	26	−	−	−	−
PreCG, precentral gyrus	−48	0	30	74	48	3	27	101
PoCG, postcentral gyrus	−45	−6	33	18	−	−	−	−
HIP, hippocampus	−24	−33	−3	13	−	−	−	−
PHG, parahippocampal gyrus	−24	−36	−12	25	21	−33	−12	39

### Modality-Preferential Regions Identified by PSC Analysis

**FIGURE 5:**
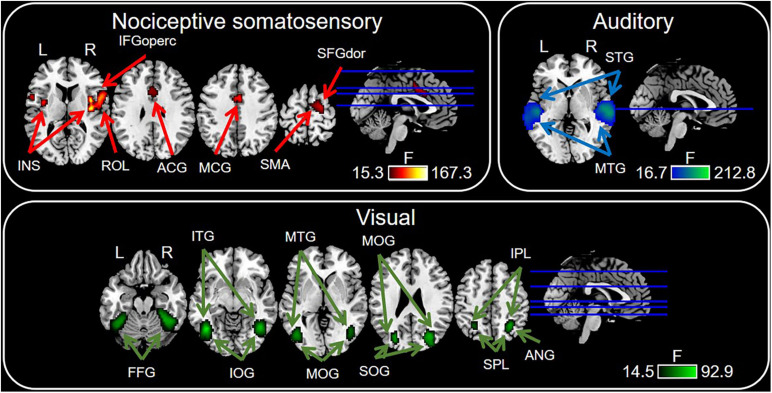
Sensory modality-preferential regions defined by voxel-wise one-way repeated measures ANOVA. Significant results were identified at *P* < 0.0001 with FDR correction. ACG, anterior cingulate gyrus; ANG, angular gyrus; MCG, middle cingulate gyrus; FFG, fusiform gyrus; IFGoperc, opercular part of inferior frontal gyrus; INS, insula; IOG, inferior occipital gyrus; IPL, inferior parietal lobule; ITG, inferior temporal gyrus; MOG, middle occipital gyrus; MTG, middle temporal gyrus; ROL, rolandic operculum; SFGdor, dorsolateral part of superior frontal gyrus; SMA, supramarginal gyrus; SOG, superior occipital gyrus; SPL, superior parietal lobule; STG, superior temporal gyrus.

Figure 5 and Table 2 showed the PSC features that are preferentially modulated by sensory modality, as revealed by voxel-wise one-way repeated measures ANOVA at *P* < 0.0001 with FDR correction, while *P* < 0.05 for the preferential brain regions see Supplementary Figure 2. The nociceptive somatosensory-preferential regions include the cingulate gyrus, frontal lobe (including the supplementary motor area, the opercular part of inferior frontal gyrus, the dorsolateral part of superior frontal gyrus), insula, and rolandic operculum. The auditory-preferential regions include the ipsilateral and contralateral temporal gyrus, and visual-preferential regions were mainly in and around the occipital lobe. However, we did not find any non-nociceptive somatosensory-preferential cortical regions.

**TABLE 2:** List of modality-preferential brain regions as revealed by percentage signal change (PSC).

Effect	Left hemisphere				Right hemisphere			
		
Region	x	y	z	No. of voxels	x	y	z	No. of voxels
**Nociceptive-somatosensory**								
ROL, rolandic operculum	−	−	−	−	39	−15	18	104
SFGdor, dorsolateral part of superior frontal gyrus	−	−	−	−	21	−9	66	56
IFGoperc, opercular part of inferior frontal gyrus	−57	9	9	25	54	9	9	38
SMA, supplementary motor area	−	−	−	−	6	0	48	94
INS, insula	−39	−6	3	17	36	−15	15	41
ACG, anterior cingulate gyrus	−3	15	30	15	−	−	−	−
MCG, middle cingulate gyrus	0	3	42	32	3	5	39	38
**Auditory**								
STG, superior temporal gyrus	−51	−27	6	302	63	−24	6	470
MTG, middle temporal gyrus	−63	−33	9	185	63	−33	6	87
**Visual**								
SOG, superior occipital gyrus	−24	−75	27	15	27	−69	30	86
MOG, middle occipital gyrus	−42	−69	0	170	27	−72	30	204
IOG, inferior occipital gyrus	−42	−63	−6	98	42	−60	−12	18
FFG, fusiform gyrus	−42	−54	−15	129	36	−48	−21	171
SPL, superior parietal lobule	−24	−60	45	21	30	−60	51	15
IPL, inferior parietal lobule	−27	−57	42	19	30	−54	48	24
ANG, angular gyrus	−	−	−	−	30	−54	45	36
MTG, middle temporal gyrus	−42	−63	−3	14	42	−63	−3	59
ITG, inferior temporal gyrus	−42	−60	−9	85	48	−51	−15	169

Further, we list the overlaps between modality-preferential regions and modality-specific regions in Table 3. It can be seen that PSC-based modality-preferential regions and GLM-based modality-specific regions were partially similar, though some differences existed. For example, we can find non-nociceptive-specific regions but cannot find non-nociceptive-preferential regions. According to the definitions of nociceptive-specific and -preferential regions, nociceptive-specific regions should be a subset of nociceptive- preferential regions.

**TABLE 3:** Common regions of modality-specific regions and modality-preferential regions.

Region	No. of voxels	Common regions					
		
		Modality-specific regions			Modality-preferential regions		
			
		x	y	z	x	y	z
**Nociceptive-somatosensory**							
SFGdor_R, dorsolateral part of superior frontal gyrus	36	21	−12	63	21	−9	63
SMA_R, supplementary motor area	24	15	−9	66	15	−6	69
SMA_R, supplementary motor area	32	6	3	51	6	0	48
**Auditory**							
STG_L, superior temporal gyrus	5	−54	−9	−3	−54	−9	−3
STG_L, superior temporal gyrus	6	−60	−21	3	−60	−24	3
STG_L, superior temporal gyrus	17	−45	−33	6	−45	−33	6
STG_R, superior temporal gyrus	29	63	−15	3	63	−21	3
MTG_L, middle temporal gyrus	40	−51	−33	6	−54	−27	3
**Visual**							
SOG_L, superior occipital gyrus	15	−24	−66	27	−24	−75	33
SOG_R, superior occipital gyrus	67	30	−63	39	30	−69	39
MOG_L, middle occipital gyrus	88	−24	−63	39	−42	−69	0
MOG_R, middle occipital gyrus	98	30	−63	36	27	−72	30
FFG_L, fusiform gyrus	5	−27	−42	−18	−30	−39	−21
FFG_R, fusiform gyrus	48	21	−33	−15	36	−48	−21
SPG_L, superior parietal lobule	20	−27	−60	45	−24	−60	45
IPL_L, inferior parietal lobule	17	−27	−54	42	−27	−54	42
IPL_R, inferior parietal lobule	19	33	−54	45	30	−54	48
ANG_R, angular gyrus	31	33	−57	51	27	−54	45
MTG_L, middle temporal gyrus	12	−45	−57	−3	−42	−63	−3
MTG_R, middle temporal gyrus	52	42	−69	18	42	−63	−3
ITG_R, inferior temporal gyrus	92	48	−48	−15	48	−51	−15

We tested for correlation between VAS ratings and PSCs for the modality-preferential regions. The correlation analysis revealed a significant correlation for visual (*P* = 0.0042) and nociceptive somatosensory (*P* = 1.34268 × 10^–9^), and a marginally significant correlation for auditory (*P* = 0.0536).

## Discussion

In this study, we conducted a multisensory fMRI experiment on a relatively large number of subjects (80 healthy participants), with the aim to identify nociceptive-specific regions and nociceptive-preferential brain regions. By using GLM and conjunction analysis, we identified a series of modality-specific regions, which were in response to one certain type of sensory modality only. In addition, by using PSC features and ANOVA, we identified a series of modality-preferential regions, which preferentially responded to a certain sensory modality, instead of simply following the conventional concept of sensory-specific regions.

### Sensory Modalities Used in This Study

In this work, we used four types of sensory stimuli, nociceptive somatosensory, non-nociceptive somatosensory, auditory, and visual, aiming to identify nociceptive-specific or -preferential cortical regions. Previous studies (Baliki et al., 2009; Mouraux and Iannetti, 2009; Mouraux et al., 2011; Liberati et al., 2016) have repeatedly found that, nociceptive-somatosensory-evoked brain regions are not only overlapped with non-nociceptive-somatosensory-evoked regions, but also overlapped with visual- or auditory-evoked regions. Importantly, there may exist some regions that are evoked by nociceptive-somatosensory and visual (or auditory), but not by non-nociceptive-somatosensory stimuli (see Supplementary Figures 3, 4, which were obtained from our data). Therefore, we used visual or auditory stimuli to rule out more false-positive results that were not real nociceptive-specific cortical regions. Actually, a perfect multisensory study to investigate modality-specific regions should include all types of sensory modalities, which is impossible in practice. Therefore, to achieve a tradeoff, we followed other classical multisensory studies (Downar et al., 2000; Mouraux et al., 2011; Morrow et al., 2019; Sanchez et al., 2020) to include visual and auditory stimuli, which are common and easy to implement, in our experiment. In addition, because the brain response patterns of visual and auditory stimuli are well-documented, the results could be used to benchmark the data analysis method used.

### Comparison Between Modality-Specific and Modality-Preferential Regions

By using the conventional GLM and conjunction analysis, we identified a set of nociceptive-specific regions, including the dorsolateral part of superior frontal gyrus, supplementary motor area, medial part of superior frontal gyrus, cingulate gyrus and precuneus. On the other hand, by based on PSC features, the identified nociceptive-preferential regions include the rolandic operculum, dorsolateral part of superior frontal gyrus, opercular part of inferior frontal gyrus, supplementary motor area, insula, anterior, and middle cingulate gyrus.

We can see from Tables 1–3 that, although the identified modality-specific and modality-preferential regions shared some common regions (as listed in Table 3), they still have remarkable difference. Theoretically, modality-specific regions should be a subset of modality-preferential regions, because one region only evoked by one type of sensory modality will have greater responses to this sensory stimulation than to other types of sensory stimulation. But our results did not agree with the above ideal and expected results. For example, we found non-nociceptive-specific regions but could not find any non-nociceptive-specific regions. It is because that the methods used to identify modality-specific and modality-preferential regions are different and it is not fair to directly compare modality-specific and modality-preferential regions identified in this work. Actually, we followed the experimental design and data analysis procedure in Mouraux et al. (2011) to check the existence of modality-specific regions. Our findings are in general consistent with the results reported in Mouraux et al. (2011). However, because this study has much more participants, we can identify some nociceptive-specific regions but Mouraux et al. (2011) did not. To further identify modality-preferential regions, we used a new data analysis pipeline (PSC analysis). As a consequence, the modality-specific and modality-preferential regions identified in the present study are not directly comparable.

### Functional Roles of Nociceptive-Preferential Regions

In the following, we mainly discussed a set of important brain regions identified as nociceptive-preferential regions. For other sensory modalities, their specific or preferential regions are highly consistent with literature, so these regions are not discussed here.

#### Posterior Insula

We observed that in the insula, multimodal regions (i.e., voxels activated by nociceptive somatosensory, non-nociceptive somatosensory, auditory, and visual regardless of the sensory modality) and nociceptive somatosensory-specific regions included the anterior insula, while nociceptive somatosensory-preferential regions included the anterior and posterior insula. This finding is consistent with previous results supporting that the posterior insula is involved in the processing of pain perception and nociceptive sensory input (Segerdahl et al., 2015; Wu et al., 2017; Liberati et al., 2019), and this region can cause pain when it is directly electrically stimulated (Mazzola et al., 2009, 2012, 2019). Our results showed that the posterior insula was nociceptive-preferential, but not specific for nociceptive somatosensory, suggesting subregions of insula may play different roles in sensory perception.

#### Supplementary Motor Area and Dorsolateral Superior Frontal Gyrus

Our results indicate that the BOLD response in the supplementary motor area and dorsolateral superior frontal gyrus was nociceptive somatosensory-specific and nociceptive somatosensory-preferential. Both supplementary motor area and dorsolateral superior frontal gyrus belong to the frontal lobe. The dorsolateral superior frontal gyrus is found to be correlated with the control of complex movements (Martino et al., 2011), and the response in the supplementary motor area is often considered to play an important role in motor control (Eccles, 1982; Tanji and Shima, 1994; Tanji, 1996). Besides, imaging movements of a hand without performing, it can also activate the supplementary motor area (Lotze et al., 1999; Al-Wasity et al., 2021). Therefore, these brain regions related to motor perception were activated may be interpreted as the participants have a strong desire to inhibit the movement and take away their hands because of the unpleasant feeling of nociceptive somatosensory stimuli.

#### Cingulate Cortex

Nociceptive somatosensory-specific and nociceptive somatosensory-preferential regions included the cingulate gyrus, but they did not overlap. Nociceptive somatosensory-specific regions in the cingulum included the anterior and middle cingulate gyrus, while nociceptive somatosensory-preferential regions included the anterior, middle and posterior cingulate gyrus. Numerous studies repeatedly observe that the cingulate gyrus is involved in nociception (Davis et al., 1995; Dum et al., 2009; Jensen et al., 2016). The anterior cingulate cortex is involved in pain processing (Devinsky et al., 1995; Hutchison et al., 1999; Fulbright et al., 2001), the middle cingulate cortex is correlated with the attentional orienting to pain (Peyron et al., 2000), and the posterior cingulate cortex is thought to be correlated with pain memory (Bromm, 2001). These results and our results showed different roles of different parts of cingulate gyrus in the processing of sensory inputs.

### Limitation and Future Work

Our results are different from the previous study (Mouraux et al., 2011), which claimed that nociceptive-specific regions do not exist. Such a difference may be due to the difference of the number of participants [14 participants in the previous study (Mouraux et al., 2011) and 80 participants in the present study] and the difference of experimental design. On the other hand, this study is still not sufficient to ascertain the existence of nociceptive-specific regions because of the following three reasons. First, the number of participants was still limited. Second, the experimental design did not cover all possible types of simulation and all possible stimulation intensity. We only used four types of stimuli (visual, auditory, non- nociceptive somatosensory, and nociceptive somatosensory) in the experiments. It is almost impossible to exhaust the full range of sensory modalities, so this work is a limited but feasible attempt. Also, we believe that the real nociceptive-specific cortical regions might be a subset of the nociceptive -specific regions identified in this study, because (1) both real and identified specific regions can respond to one sensory modality, (2) real specific regions do not respond to all other possible sensory modalities while identified specific regions do not respond to a small number of other sensory modalities included in the experiment. Third, these identified nociceptive-specific regions were sometimes reported to be respond to various types of stimulation and tasks in literature. It is always difficult to claim one brain region is specific to one type of stimulation or task, because normally a brain region has multiple functions. Anyhow, we believe this study is still an important step to advance our understanding of human brain’s processing of different types of sensory modalities.

## Conclusion

In this study, we conducted a multisensory fMRI experiment on 80 healthy participants, with the aim to determine whether there are certain brain regions that specifically or preferentially respond to nociceptive stimulation. We found a series of brain responses involved in nociceptive sensory input. Specifically, we identified nociceptive-specific regions including the dorsolateral part of superior frontal gyrus, supplementary motor area, medial part of superior frontal gyrus, cingulate gyrus and precuneus, and nociceptive-preferential regions including the rolandic operculum, dorsolateral part of superior frontal gyrus, opercular part of inferior frontal gyrus, supplementary motor area, insula, anterior and middle cingulate gyrus. The nociceptive somatosensory specific and preferential regions may play a vital role in the processing of nociceptive sensory input.

## Data Availability Statement

The datasets presented in this article are not readily available due to the nature of this research, participants of this study did not agree for their data to be shared publicly, so supporting data is not available. Requests to access the datasets should be directed to ZZ, zgzhang@szu.edu.cn.

## Ethics Statement

The studies involving human participants were reviewed and approved by the Ethics Committee of Institute of Psychology, Chinese Academy Sciences, the Ethics Committee of Liaoning Normal University, and the Ethics Committee of Shenzhen University. Written informed consent to participate in this study was provided by the participants’ legal guardian/next of kin. Written informed consent was obtained from the individual(s), and minor(s)’ legal guardian/next of kin, for the publication of any potentially identifiable images or data included in this article.

## Author Contributions

ZZ, LL, and GH conceived of the presented idea. XZ developed the theory and performed the computations. ZL, LS, and LZ supervised the findings of this experiment. All authors discussed the results and contributed to the final manuscript.

## Conflict of Interest

The authors declare that the research was conducted in the absence of any commercial or financial relationships that could be construed as a potential conflict of interest.

## Footnotes

1 www.fil.ion.ucl.ac.uk/spm/software/spm12
