# Supplementary material for: A Multisensory fMRI Investigation of Nociceptive-Preferential Cortical Regions and Responses
Source: Front Neurosci. 2021 Apr 14;15:635733. doi: 10.3389/fnins.2021.635733 (PMC8079658; doi:10.3389/fnins.2021.635733)
Supplement: Supplementary file 1 [file Presentation_1.pdf]

Supplementary Material

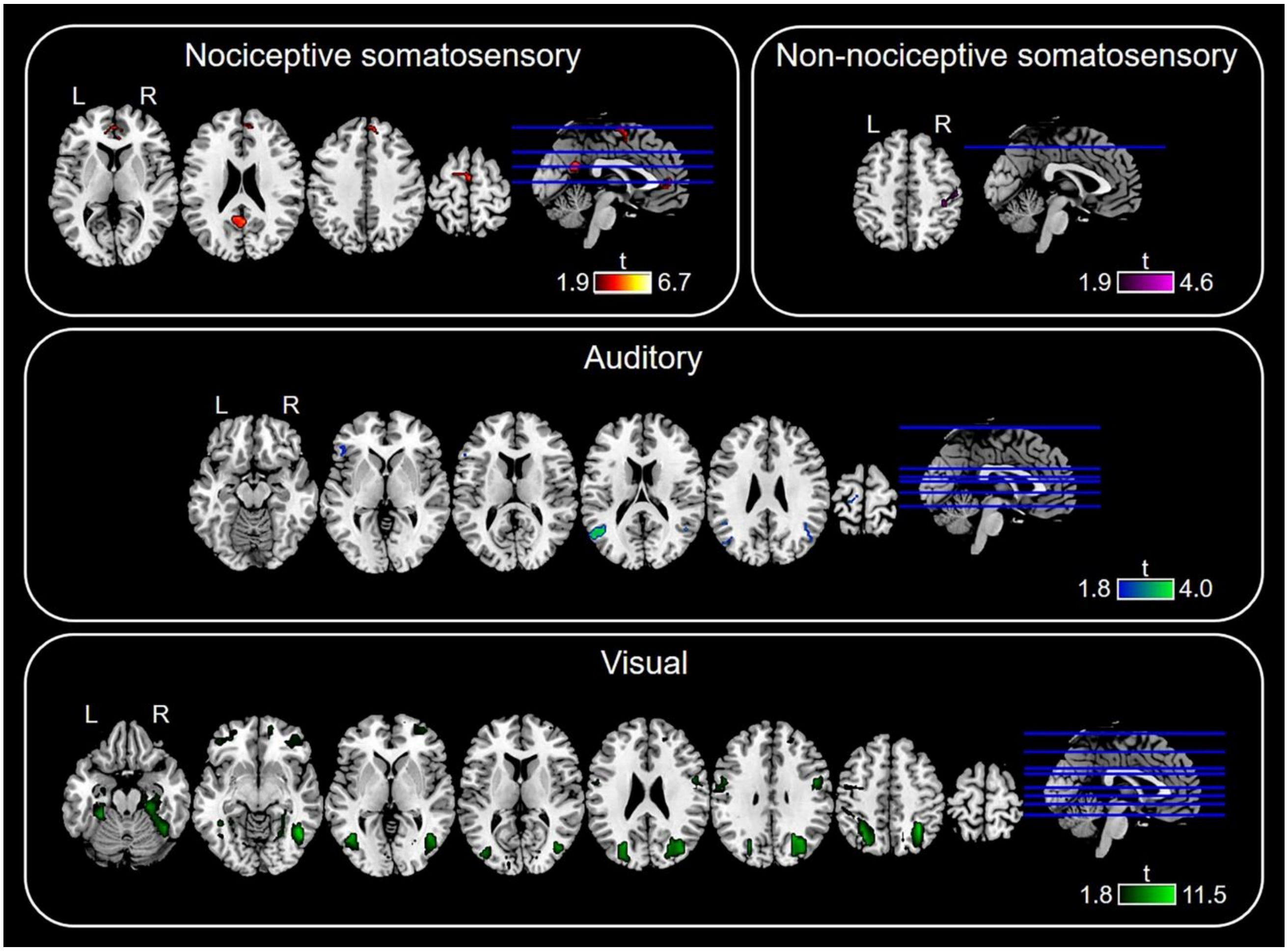

Supplementary Figure S1. Sensory modality-specific regions defined by conjunction analysis of GLM maps. Significant results were identified at P<0.05 with FDR correction.

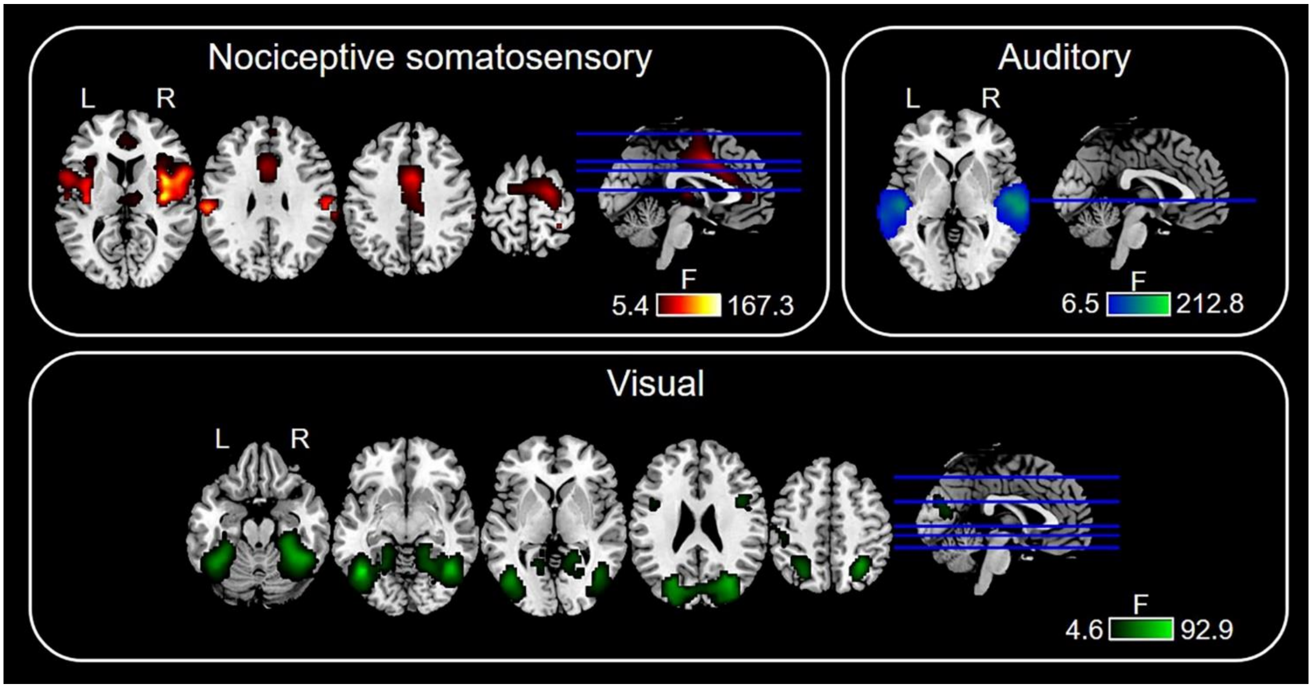

Supplementary Figure S2. Sensory modality-preferential regions defined by voxel-wise one-way repeated measures ANOVA. Significant results were identified at P<0.05 with FDR correction.

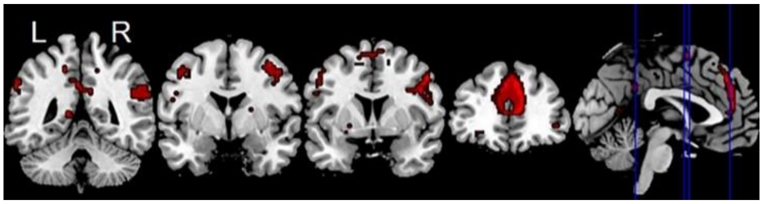

Supplementary Figure S3. Binary brain regions activated by nociceptive-somatosensory and visual, but not by non-nociceptive-somatosensory stimulation. Significant results were defined by conjunction analysis of GLM maps and identified at P<0.0001 with FDR correction.

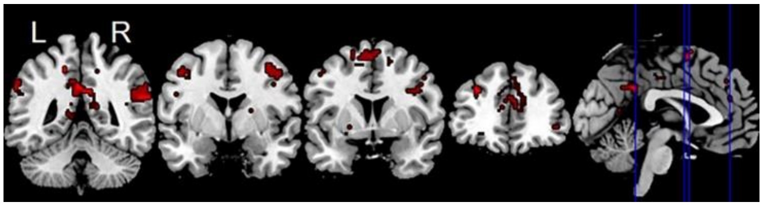

Supplementary Figure S4. Binary brain regions activated by nociceptive-somatosensory and auditory, but not by non-nociceptive-somatosensory stimulation. Significant results were defined by conjunction analysis of GLM maps and identified at P<0.0001 with FDR correction.
